# Assessment of Multifunctional Activity of a Postbiotic Preparation Derived from *Lacticaseibacillus paracasei* Postbiotic-P6

**DOI:** 10.3390/foods13152326

**Published:** 2024-07-24

**Authors:** Hui Dong, Xianpu Ren, Yaxin Song, Jingwen Zhang, Haonan Zhuang, Chuantao Peng, Jinshan Zhao, Jinling Shen, Jielin Yang, Jinhong Zang, Day Li, Tanushree B. Gupta, Dehua Guo, Zhaojie Li

**Affiliations:** 1 School of Food Science and Engineering, Qingdao Agriculture University, Qingdao 266000, China; dong1789611821@163.com (H.D.); kxklrxp@163.com (X.R.); syx001004@163.com (Y.S.); 17861218720@163.com (J.Z.); 17660433075@163.com (H.Z.); chuantaopeng@163.com (C.P.); zhaojinshan@qau.edu.cn (J.Z.); 15190273775@163.com (J.Z.); 2 Special Food Research Institute, Qingdao Agricultural University, Qingdao 266000, China; 3 Technology Center for Animal Plant and Food Inspection and Quarantine, Shanghai Customs, Shanghai 200000, China; jinling_zhan19@163.com (J.S.); yangjielin@customs.gov.cn (J.Y.); guodehua@customs.gov.cn (D.G.); 4 Food System Integrity Team, Hopkirk Research Institute, AgResearch, Palmerston North 4474, New Zealand; li.day@agresearch.co.nz (D.L.); tanushree.gupta@agresearch.co.nz (T.B.G.)

**Keywords:** postbiotic, *Lacticaseibacillus paracasei* Postbiotic-P6, antihemolytic activity, anti-inflammatory activity, antioxidant activity, antibacterial activity, functional food supplement, food preservation

## Abstract

Postbiotics possess various functional activities, closely linked to their source bacterial strains and preparation methods. Therefore, the functional activities of postbiotics need to be evaluated through in vitro and in vivo methods. This study aims to prepare a postbiotic and explore its antihemolytic, anti-inflammatory, antioxidant, and antibacterial activities. Specifically, a postbiotic preparation named PostbioP-6 was prepared by intercepting 1–5 kDa of *Lacticaseibacillus paracasei* Postbiotic-P6 fermentation broth. The results demonstrate that PostbioP-6 exhibited notable biological activities across multiple assays. It showed significant antihemolytic activity, with a 4.9–48.1% inhibition rate at 10–50% concentrations. Anti-inflammatory effects were observed both in vitro, where 8–40% PostbioP-6 was comparable to 259.1–645.4 μg/mL diclofenac sodium, and in vivo, where 3.5 and 4.0 μL/mL PostbioP-6 significantly reduced neutrophil counts in inflamed zebrafish (*p* < 0.05). Antioxidant properties were evident through increased reducing power (OD_700_ increased from 0.279 to 2.322 at 1.25–12.5% concentrations), DPPH radical scavenging activity (38.9–92.4% scavenging rate at 2.5–50% concentrations), and hydroxyl radical scavenging activity (4.66–10.38% scavenging rate at 0.5–4% concentrations). Additionally, PostbioP-6 demonstrated antimicrobial activity against two Gram-positive bacteria, eight Gram-negative bacteria, and one fungus. Furthermore, PostbioP-6 significantly inhibited the increase in peroxide value and malondialdehyde content in cookies, highlighting its potential application in food preservation. In conclusion, we prepared a novel postbiotic, termed PostbioP-6, which proved to have prominent anti-hemolytic, anti-inflammatory, antioxidant, and broad-spectrum antimicrobial activities. The multifunctional properties of PostbioP-6 position it as a potentially effective functional food supplement or preservative. In the future, further research is necessary to elucidate the precise mechanisms of action, identify the active components, and validate its biological activities in animal models or clinical trials.

## 1. Introduction

The use of beneficial microorganisms, termed probiotics, in healthcare domains has a long history. Despite the beneficial functions of probiotics, recent studies have shown that there are challenges to the use of these bacteria. One of the challenges is their ability to survive in complicated food matrixes or in vivo gastrointestinal tracts. The harsh conditions of the gastrointestinal tract, including the acidic stomach environment, bile salts in the small intestine, and various digestive enzymes, may reduce the number of active probiotic cells. The ability of probiotics to colonize the intestinal tract remains a major challenge [1]. Additionally, food processing techniques, such as heat treatment, refrigeration, and drying, can harm bacteria, affecting the survival of probiotics [2]. The potential influence of non-viable bacterial cells and their components/metabolites on probiotic functionality has gained little attention. Another important challenge is the safety concerns with the use of probiotics by certain groups, such as neonates [3,4] and vulnerable populations [5]. Some studies have reported that the intake of probiotics could result in microbial translocation, infections, and inflammation [6,7]. More importantly, given the shared gastrointestinal tract environment, there is a risk of transmitting antibiotic resistance genes through pathogenic microorganisms to other commensal probiotics and vice versa [8]. In recent years, researchers have proposed various solutions to address these challenges. Notably, using inactive bacterial cells or their components/metabolites is an effective way to overcome these challenges, making them a viable alternative to active probiotics [9,10,11,12,13,14].

In 2021, the International Scientific Association of Probiotics and Prebiotics (ISAPP) proposed the definition of the term postbiotic: “a preparation of inanimate microorganisms and/or their components that confers a health benefit on the host” [9]. Postbiotics, which contain varying biologically active components, have been extensively linked with improvements in nutrition and health. Especially in recent years, there has been growing evidence that postbiotics are useful tools in the fight against human diseases, and the focus of current research is gradually shifting from live probiotics to postbiotics. For instance, numerous in vitro and in vivo experiments have shown that postbiotics can play a positive role in boosting immunity [10], preventing colon cancer [11], maintaining oral health [12], preventing osteoporosis [13], and combating allergies [14], etc. After inactivation, primarily through heat treatment, dead bacterial cells can release many bioactive components with key immunomodulatory and antibacterial effects. Components, such as peptidoglycans, exopolysaccharides (EPS), and lipoteichoic acids, are chiefly responsible for these properties [15,16]. Additionally, postbiotics are preferred over probiotics because of their safety, nontoxicity, resistance to pH and temperature, resistance to gut enzymes, stability in the gastrointestinal tract, and the reduced risks related to live microorganisms, such as microbial translocation, infection, or aggravated inflammation [15,17].

Undoubtedly, the health benefits and advantages of postbiotics will promote their study and practical application in healthcare. However, it is known that different types of postbiotics may have different functions, which might be due to their varying composition. This also implies that the health benefits of postbiotics may be driven by different mechanisms [9]. The components of a postbiotic can be cellular components or metabolites or their inactivated cells, such as peptidoglycans, exopolysaccharides (EPS), lipoteichoic acids, bacteriocins, short-chain fatty acids, organic acids, and some biosurfactants [9,18]. Izuddin prepared a cell-free supernatant (CFS) of *Lactobacillus plantarum* RG14 and explored its positive effects on the growth performance, rumen microbial profile, and blood metabolites in post-weaning lambs [19]. Kwon reported that exopolysaccharides from *Lactobacillus plantarum* L-14 had anti-inflammatory effects via the toll-like receptor 4 pathway in LPS-induced RAW 264.7 cells [20]. Wuri found that both live *Lactobacillus paracasei* ET-22 and its heat-killed bacterial cells could modulate oral microbiome dysregulation and reduce halitosis [21]. Moreover, taking the example of the antibacterial activity of postbiotics against *Streptococcus mutans*, biosurfactants significantly inhibited biofilms, lipoteichoic acids exhibited anticaries capabilities, metabolites from *L. fermentum* TcUESC01 inhibited adherence and displayed bactericidal activities on *S. mutans* planktonic cells, and organic acids demonstrated bacteriostatic activity on *S. mutans* [18]. Given the multi-component properties of postbiotics, it should be noted that such modes of action might act independently or in combination. It can be concluded that the health effects of postbiotics are typically specific to the bacterial strain. In other words, postbiotics derived from different bacterial strains or prepared through various processes may exhibit distinct functions. The general characteristics and health effects of a postbiotic need to be first evaluated using both in vitro and in vivo methods.

Postbiotics with a single activity, such as antibacterial, antioxidant, or immunomodulatory effects, or those targeting a specific disease, like irritable bowel syndrome, non-alcoholic fatty liver disease, and gout, have been well documented [22,23,24,25]. However, few studies demonstrate the simultaneous multifunctional activities of postbiotics. Such multifunctional properties may be preferred over single-activity postbiotics, as they can provide comprehensive health promotion by concurrently triggering, modulating, or inhibiting multiple physiological pathways. Therefore, it is of great significance to investigate the multifunctional properties of postbiotics simultaneously, which will enhance their application in healthcare.

*Lacticaseibacillus paracasei* Postbiotic-P6 (*L. paracasei* Postbiotic-P6)*,* was obtained from the Lactic Acid Bacteria Collection Center at Qingdao Agricultural University. The aim of this study is to prepare a postbiotic from *L. paracasei* Postbiotic-P6, and comprehensively investigate its multifunctional properties, including anti-hemolytic, anti-inflammatory, antioxidant, and antimicrobial activities, using both in vitro and in vivo methods. Meanwhile, we expect to explore the potential of the postbiotic in inhibiting food peroxidation. This study will help to promote the application of postbiotics in the food and pharmaceutical fields.

## 2. Materials and Methods

### 2.1. Bacterial Strains and Reagents

*Lacticaseibacillus paracasei* Postbiotic-P6 (*L. paracasei* Postbiotic-P6) was obtained from the Lactic Acid Bacteria Collection Center at Qingdao Agricultural University and deposited in the General Microbiology Center of the China Microbial Strain Deposit Management Committee, with the deposit number CGMCC 26237. *L. paracasei* Postbiotic-P6 was activated in Man–Rogosa–Sharpe (MRS, Qingdao HopeBio Co., Qingdao, China) liquid media at 37 °C for 24 h, aerobically. All the indicator bacteria were activated in Luria-Bertani broth (LB, Qingdao HopeBio Co., Qingdao, China) at 37 °C for 24 h and all the fungi were activated in potato dextrose broth (PDB, Qingdao HopeBio Co., Qingdao, China) at 30 °C for 48 h. Mouse erythrocyte solution was purchased from Jiangsu Kewei Biotechnology Co. (Nantong, China). All the other chemicals used in this study were analytically pure and obtained domestically.

### 2.2. Preparation of PostbioP-6 and Determination of Biogenic Amine Production

The PostbioP-6 was prepared according to the method described in [14], with some modifications. Revived *L. paracasei* Postbiotic-P6 was inoculated into the MRS medium (2%, *v*/*v*) and cultured at 37 °C for 24 h. Then, the bacterial suspension was inactivated at 121 °C for 15 min. The inactivated suspension was ultrasonicated in an ice bath at 300 W, with cycles of 10 s on/10 s off, for 15 min. A microscopic examination confirmed the absence of intact bacteria. The suspension was then centrifuged (4 °C, 6000× *g*) for 10 min to remove bacterial fragments. The cell-free supernatant (CFS) was filtered through a 0.22 μm membrane. The *L. paracasei* Postbiotic-P6 CFS was desalted using electrodialysis equipment (Guochu Technology, Xiamen, China), until the electrical conductivity reached 3.0 ms/cm. The desalted CFS was fractionated using ultrafiltration membranes, with molecular weight cutoffs of 1 and 5 kDa (Guochu Technology, Xiamen, China). The 1–5 kDa ultrafiltrate was collected, stored at −20 °C, and named PostbioP-6.

To confirm the safety of PostbioP-6, nine biogenic amines (BA) in PostbioP-6, namely tryptamine, phenylethylamine, putrescine, cadaverine, histamine, octopamine, tyramine, spermidine, and spermine, were determined according to the method described in [26]. Quantitative determination of the biogenic amines was performed using HPLC, with gradient elution. Solvent A consisted of 90% acetonitrile and 10% 10 mmol/L ammonium acetate, while solvent B consisted of 10% acetonitrile and 90% 10 mmol/L ammonium acetate. The gradient program was: 60% A initially, increased to 85% (0–22 min), then to 100% (22–25 min), and held for 7 min, then decreased to 65% over 5 min. The flow rate was 0.8 mL/min, with UV detection at 254 nm. An injection volume of 20 μL was used. The contents of the biogenic amines were quantified by comparing the sample peak areas to those of the biogenic amine standard.

### 2.3. Antihemolytic Activity Assay

The antihemolytic activity of PostbioP-6 was determined according to the method by Elkolli, with minor modifications [24]. PostbioP-6 was diluted to final concentrations of 40%, 32%, 24%, 16%, and 8%, using distilled water. Aliquots of 500 µL of each diluted sample were mixed with 500 μL of mouse erythrocyte suspension. The mixtures were incubated in a water bath at 56 °C for 30 min to induce hemolysis. After incubation, the samples were immediately cooled on ice, centrifuged (2500× *g*, 5 min, 4 °C), and the absorbance of the supernatants was measured at 575 nm. An equal volume of isotonic solution (0.9% NaCl), incubated with mouse erythrocyte suspension and treated identically to the PostbioP-6 samples, served as the positive control. An equal volume of isotonic solution, mixed with the mouse erythrocyte suspension but not heated, served as the negative control. The percentage of inhibition of hemolysis was calculated by the following equation:

(1) $$X=\frac{A-B}{C-B}\times 100\%$$

in the formula:

*X*—hemolysis rate;

*A*—absorbance of the test group;

*B*—negative control group absorbance;

*C*—positive control group absorbance.

### 2.4. In Vitro and In Vivo Anti-Inflammatory Activity Tests

The in vitro anti-inflammatory activity of PostbioP-6 was evaluated using the albumin denaturation inhibition method, as described by Chandra, with minor modifications [27]. Specifically, the PostbioP-6 solutions at concentrations of 100%, 80%, 60%, 40%, and 20% were prepared with distilled water. Each solution (250 μL) was mixed with 25 μL of egg white protein from fresh eggs and 350 μL of phosphate-buffered saline (PBS, pH 7.2, 0.01 M), in separate tubes. The mixtures were incubated at 37 °C for 15 min, followed by denaturation at 70 °C for 5 min. The tubes were then rapidly cooled on ice for 5 min and 200 μL of each sample was transferred to a 96-well flat-bottomed plate. The absorbance was measured at 660 nm. Distilled water was used as the negative control and various concentrations of diclofenac sodium served as references to evaluate the anti-inflammatory activity of PostbioP-6. The inhibition percentage of the protein denaturation was calculated using the following formula:

(2) $$X=\left(\frac{A}{B}-1\right)\times 100$$

in the formula:

*X*: inhibition rate of the protein denaturation;

*A*: absorbance of the sample;

*B*: absorbance of the control group.

Additionally, the in vivo anti-inflammatory activity was assessed using a transgenic (*mpx*-EGFP) zebrafish inflammation model, following the method by He, with some modifications [28]. At 3 days post-fertilization (dpf), zebrafish larvae (n = 12) were randomly distributed into 24-well plates and categorized into one negative control group (NC) and nine CuSO4-treated groups. These nine CuSO_4_-treated groups included: one group pretreated with CuSO_4_ only, one group pretreated with 20 μM indomethacin, and seven groups pretreated with PostbioP-6 at concentrations of 0.1%, 0.15%, 0.2%, 0.25%, 0.3%, 0.35%, and 0.4%. Following a 2 h pretreatment at 28 °C with indomethacin or PostbioP-6, the CuSO_4_-treated groups were exposed to 60 μM CuSO_4_ for 2 h at 28 °C. The larvae were then rinsed, anesthetized, and imaging was carried out using a fluorescence microscope. Image-Pro Plus 5.1 software was used to quantify the number of neutrophils at the inflammation site in the zebrafish larvae.

### 2.5. In Vitro Antioxidant Activity Assessment

Antioxidants have garnered significant attention for their protective roles against oxidative deterioration and in vivo oxidative stress-mediated pathological processes in food and pharmaceutical products [29]. These molecules can safely interact with free radicals, halting chain reactions by donating an electron, and transforming free radicals into harmless molecules. Herein, the antioxidant activity of PostbioP-6 was evaluated in regard to three different dimensions: reducing power, DPPH free radical scavenging activity, and hydroxyl radical scavenging activity.

### 2.6. Determination of Reducing Power

Based on the methodology by Zeng [30], appropriate modifications were made for the present study. Serial concentrations of PostbioP-6 solutions (100%, 50%, 40%, 30%, 20%, and 10%) were prepared to obtain final concentrations of 12.5%, 10%, 7.25%, 5%, 2.5%, and 1.25%, respectively. To each test tube, 0.5 mL of the different concentrations of PostbioP-6 was added, followed by 0.5 mL of PBS (pH 7.2) and 0.5 mL of 1% aqueous potassium ferricyanide. The mixtures were incubated in a water bath at 50 °C for 20 min and then rapidly cooled on ice. Subsequently, 0.5 mL of trichloroacetic acid solution (10%, *w*/*v*) was added and the supernatant was collected by centrifugation (2000× *g*, 5 min). A 1.0 mL aliquot of the supernatant was mixed with 1.0 mL of 0.1% ferric chloride solution, shaken in a vortex mixer for 10 min, and 200 μL was transferred to a 96-well plate. The absorbance at 700 nm was measured. Different concentrations of VC were used as controls and PBS served as the blank control. The reducing power was calculated according to the following formula:

(3) $$reducibility\left(\%\right)=\frac{\;A_{sample}-A_{blank}}{A_{blank}}\times 100\%$$

*A_blank_* is the absorbance of the control using PBS instead of PostbioP-6.

### 2.7. Measurement of DPPH Free Radical Scavenging Activity

The DPPH free radical scavenging capacity of PostbioP-6 was determined according to the method in [31], with appropriate modifications. DPPH was dissolved in ethanol to achieve a concentration of 0.2 mmol/L. Subsequently, 1 mL of this 0.2 mmol/L DPPH solution was added to 1 mL of PostbioP-6 at various concentrations (100%, 80%, 60%, 40%, 20%, and 10%). The mixtures were homogenized and incubated at 37 °C for 30 min in the dark. After incubation, the samples were centrifuged at 7000× *g* for 10 min. The absorbance of the supernatant was then measured at 517 nm. The DPPH clearance rate was calculated using the following formula:

(4) $$clearance\;rate\left(\%\right)=\left[1-\frac{A_{sample}-A_{0}}{A_{blank}}\right]\times 100\%\;\;$$

where *A_blank_* is the absorbance of the control using an equal volume of distilled water instead of PostbioP-6 and *A*_0_ is the absorbance of the control using an equal volume of distilled water instead of DPPH.

### 2.8. Measurement of Hydroxyl Radical Scavenging Activity

The hydroxyl radical scavenging activity of PostbioP-6 was measured using a hydroxyl radical scavenging activity assay kit (Solarbio, Beijing, China), according to the manufacturer’s instruction. After the reaction, the absorbance at 536 nm was measured. The hydroxyl radical scavenging rate was measured according to the instructions.

### 2.9. Antibacterial Activity

The antimicrobial activity of PostbioP-6 was evaluated following the method by Setyo Putri et al., with appropriate modifications [32]. The PostbioP-6 was centrifuged at 8000× *g* for 10 min and the supernatant was filtered through a 0.22 μm membrane prior to use. The activated indicator bacteria or fungi were adjusted to a concentration of 10^7^ CFU/mL with PBS, mixed with sterilized molten nutrient agar (1%, *v*/*v*), and poured (20 mL per assay) into sterile plates. After the medium solidified, 8 mm diameter wells were punched into the agar. Each well was filled with 200 μL of PostbioP-6 or PBS, in triplicate. The plates were incubated at 4 °C for 4 h to allow diffusion, followed by incubation at 37 °C for 12 h for the bacteria, and at 30 °C for 48 h for the fungi. The size of the inhibition zones was then measured. PBS was used as a negative control.

### 2.10. The Effects of PostbioP-6 on the Peroxide Values and Malondialdehyde Content in Cookies

Based on the antioxidant results, different concentrations of PostbioP-6 (1%, 2%, 3%, 4%, and 5%) were added to the raw materials for making cookies. After baking, the peroxide values and malondialdehyde content in the cookies were measured. The antioxidant values of the cookies were determined according to the method outlined by Cirlini et al. (2012) [33]. Specifically, 0.1 g of the cookies was weighed in a 100 mL conical flask, to which 20 mL of methylene chloride/glacial acetic acid solution (2/3, *v*/*v*) was added. After complete dissolution of the sample, 1 mL of saturated potassium iodide solution was added. The solution was then stored in the dark at 25 °C for 16 h. Following this, 20 mL of distilled water was added to the solution, which was titrated with 0.05 M sodium thiosulfate solution, using 1 mL of starch solution as an indicator. The peroxide value was calculated according to the following equation:

(5) $$X=\left(V-V_{0}\right)\times C\times 0.1269\times m\times 100\%$$

In the formula: *X* is the peroxide value, *V* is the volume of the standard solution of sodium sulfate consumed by the samples, *V*_0_ is the volume of the standard solution of sodium sulfate consumed by the blank tests, *C* is the concentration of the standard solution of sodium sulfate, and m is the sample weight.

The malondialdehyde content in cookies was determined following the method, with minor adjustments, as outlined by Castrejón and Yatsimirsky [34]. Specifically, 2.0 mL of 0.03 M thiobarbituric acid (TBA) was mixed with 2.0 mL of each sample and incubated at 94 °C for 15 min. After cooling, the absorbance at 532 nm was measured. A calibration curve was constructed based on the absorbance of the mixtures containing 2.0 mL of 0.1–10 µM malondialdehyde working solution in 0.1 M perchloric acid and 2.0 mL of 0.03 M TBA solution. The malondialdehyde content in the samples was calculated using the following formula:

(6) $$X=\frac{C\times V}{m}$$

In the formula: *X* is the malondialdehyde content of the samples, *C* is the malondialdehyde concentration in the sample solution obtained from the standard curve, *V* is the constant volume of the sample solution, and m is the weight of the sample.

### 2.11. Statistical Analysis

All the assays were performed in triplicate. Student’s *t*-test was performed using SPSS version 19.0. The polynomial regression analysis, generalized linear logistic modelling, and allometric modeling were performed using Origin 2024. Polynomial regression analysis was conducted to examine the relationship between the concentration of PostbioP-6 and its antihemolytic, anti-inflammatory, and reducing power activities. A generalized linear logistic model was utilized to evaluate the association between the PostbioP-6 concentration and its DPPH free radical scavenging activity. Additionally, an allometric model was applied to assess the relationship between the PostbioP-6 concentration and its hydroxyl radical scavenging activity. Statistical significance was set at *p* < 0.05.

## 3. Results and Discussion

### 3.1. Determination of Biogenic Amine Production

**Figure 1:**
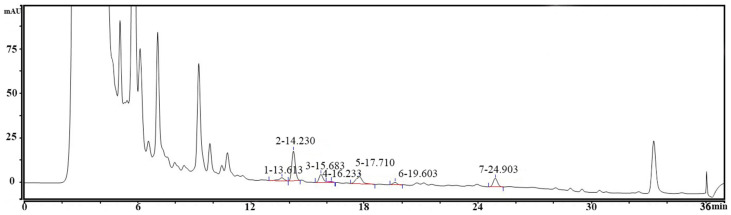
Chromatogram of 9 biogenic amines.

BAs are biologically active, low-molecular-weight organic bases, considered to be toxic in foods. Their presence often results from the decarboxylation of amino acids by microorganisms with amino carboxylase enzymes [26]. Consuming foods with high BA levels can cause migraines, nausea, sweating, hypertension, and hypotension. In lactic acid-fermented foods like cheese and fermented sausage, some species of lactic acid bacteria, particularly those producing tyramine, are notable BA producers [35]. To confirm the safety of PostbioP-6, nine BAs were analyzed. As shown in Figure 1, all nine BAs were undetectable in PostbioP-6 (below the detection limits), indicating its safety.

### 3.2. Antihemolytic Activity of PostbioP-6

**Figure 2:**
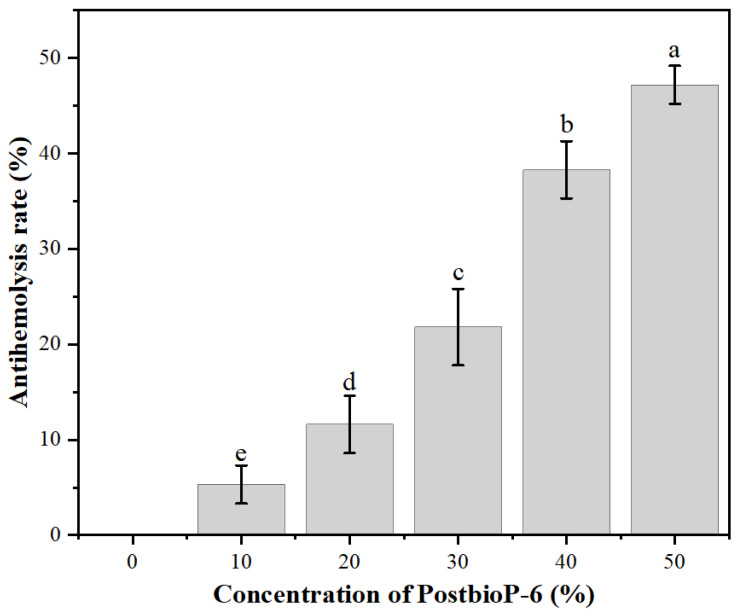
Antihemolytic activity of PostbioP-6. Different letters (a–e) indicate statistically significant differences (*p* < 0.05) between different concentrations of PostbioP-6 and the data are the mean ± SD of three replicates.

The antihemolytic assay aimed to assess whether PostbioP-6 prevented heat-induced damage to the erythrocyte membrane or not. The antihemolytic activity of PostbioP-6 is shown in Figure 2. The results show that PostbioP-6 inhibited the hemolysis of mouse erythrocytes. The incubation of erythrocytes with PostbioP-6 did not induce hemolysis, indicating its non-toxic and harmless nature. Moreover, with the increase in the PostbioP-6 concentration, the antihemolytic rate also significantly increased (*p* < 0.05). The polynomial regression analysis found that the relationship between the concentration of PostbioP-6 and its antihemolytic activity fit the equation Y = d + B_1_ × X + B_2_ × X^2^ (d = −0.45714 ± 2.23178, B_1_ = 0.46129 ± 0.20993, B_2_ = 0.0105 ± 0.00403; R^2^ = 0.98), indicating that PostbioP-6 protected the erythrocyte membrane from hemolysis in a concentration-dependent manner.

It has been reported that elevated blood temperatures can induce thermolysis of red blood cells. Diseases such as cancer, atherosclerosis, viral infections, and gout, which are associated with pyrexia (fever), can lead to erythrocyte hemolysis, by compromising the integrity of the cell membrane bilayer [35,36]. The mechanisms underlying the antihemolytic activity of postbiotics are not well understood. It has been reported that some bioactive peptides in fermented milk can enhance the stability of the erythrocyte membrane and its resistance to shear stress and can also reduce the sensitivity of the cell membrane to thermal damage, protecting erythrocytes from destruction during the hot period [37,38]. In addition, it is speculated that antioxidants, particularly polyphenols in plant extracts, protect erythrocytes from oxidant attack, by binding to the membrane matrix near tryptophan residues. This binding helps maintain erythrocyte integrity, thereby delaying hemolysis [39,40,41]. PostbioP-6 contains various active components, including polypeptides and some other active molecules. Therefore, it is reasonable to speculate that PostbioP-6 may exert its antihemolytic activity by enhancing erythrocyte membrane stability, but other mechanisms may also be involved.

### 3.3. Anti-Inflammatory Activity of PostbioP-6

**Figure 3:**
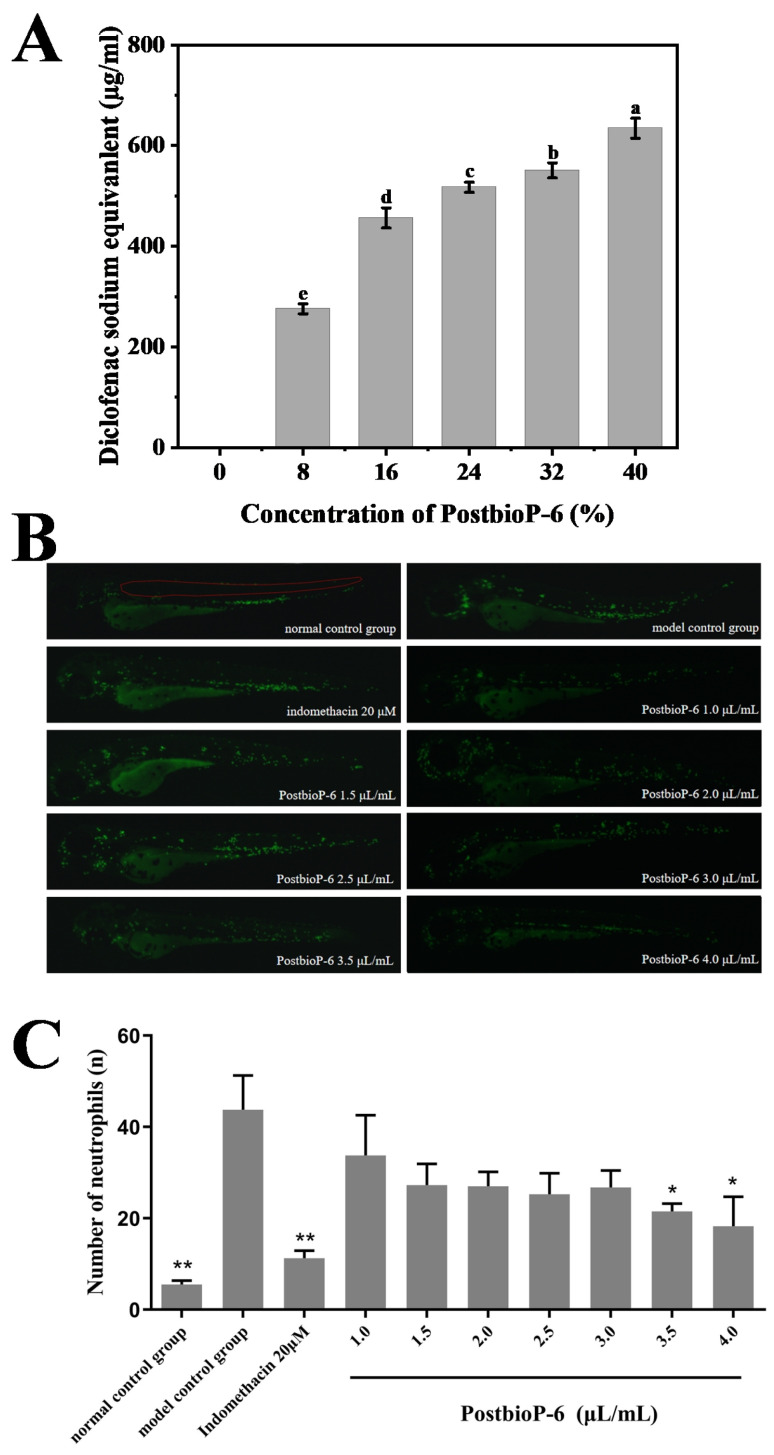
Anti-inflammatory activity of PostbioP-6. (**A**) Protein denaturation inhibition rate of PostbioP-6. Different letters (a–e) indicate statistically significant differences (*p* < 0.05) between groups. (**B**) PostbioP-6 decreased neutrophil accumulation in the lateral neural mound region. The area within the red lines is the analysis region. (**C**) Quantitative data on the anti-inflammatory efficacy of PostbioP-6 on CuSO_4_-induced zebra fish inflammation. The data are the mean ± SD of three replicates. Notes: * *p* < 0.05, ** *p* < 0.01.

During the degenerative and necrotic stages of inflammation, significant intra- and extracellular protein denaturation occurs. Therefore, the protein denaturation inhibition rate, expressed as the diclofenac sodium equivalent, can be used to evaluate the anti-inflammatory activity of a compound [36]. As shown in Figure 3A, PostbioP-6 effectively inhibited protein denaturation. The polynomial regression analysis showed that the relationship between the concentration of PostbioP-6 and its anti-inflammatory activity fit the equation Y = d + B_1_ × X + B_2_ × X^2^ (d = 23.75714 ± 40.67899, B_1_ = 31.76107 ± 4.78298, B_2_ = −0.4317 ± 0.11478; R^2^ = 0.96). Between the concentrations of 8% and 40%, with the increase in the concentration of PostbioP-6, the protein denaturation inhibition rate also significantly increased (*p* < 0.05), indicating dose-dependent anti-inflammatory activity. In particular, 8% to 40% PostbioP-6 demonstrated anti-inflammatory effects comparable to 259.1–645.4 μg/mL of the diclofenac sodium equivalent, suggesting its potential use as an anti-inflammatory drug for inflammatory diseases.

The in vivo anti-inflammatory effect of PostbioP-6 was evaluated using a Tg (mpx-EGFP) zebrafish inflammation model. As shown in Figure 3B, upon exposure to CuSO_4_, neutrophils migrated from the ventral trunk to the lateral line neural mound region, resulting in a significant increase in neutrophils in this area. Indomethacin, an anti-inflammatory drug, markedly inhibited this migration. Notably, PostbioP-6 at concentrations of 3.5 and 4.0 μL/mL significantly reduced neutrophil accumulation in the lateral neural mound region (Figure 3C, *p* < 0.05), demonstrating the potent in vivo anti-inflammatory property of PostbioP-6. Additionally, the results also confirmed the non-toxicity of PostbioP-6.

Several anti-inflammatory drugs, such as diclofenac sodium, indomethacin, atorvastatin, and prednisolone, are commonly used to treat inflammation-related diseases. However, the long-term use of conventional anti-inflammatory drugs can cause side effects. Therefore, agents that can prevent protein denaturation, especially natural substances found in food, may serve as effective adjuvants for inflammation treatment [41]. A previous study showed that milk-derived peptides have inhibitory effects on heat-induced protein denaturation and a crude extract of *L. plantarum* 55 or its fractions has high anti-inflammatory activity, with the diclofenac sodium equivalent ranging from 723.68 to 1759.43 μg/mL [36]. It has also been proved that several natural active substances, such as water lily extracts [42], Cassia oleifera extract [43], and anti-inflammatory drugs such as NSAIDs [44], can exert anti-inflammatory effects by inhibiting heat-induced protein denaturation. Obruca et al. have investigated the anti-inflammatory mechanisms and proved that some substances affected the hydrated shells around proteins and other biomolecules, thereby protecting the proteins from denaturation caused by environmental changes [45]. In addition, zebrafish and rats are the most commonly used models for in vivo anti-inflammatory assays. For instance, it has been reported that *Lacticaseibacillus rhamnosus* HN001 and *Bifidobacterium animalis* subsp. lactis Bi-07 significantly decreased neutrophil infiltration and enhanced the repair of damaged intestinal mucosa in zebrafish models of intestinal inflammation [46]. Similarly, Li demonstrated that heat-killed *Bifidobacterium longum* subsp. *infantis* B8762 could alleviate intestinal inflammation in DSS-induced IBD rats [47].

### 3.4. In Vitro Antioxidant Activity of PostbioP-6

Reactive oxygen species (ROSs) are generated through oxidation reactions within organisms. The resulting biochemical imbalance caused by ROSs can damage various biological macromolecules, including proteins, lipids, DNA, and RNA. This damage is implicated in the development of degenerative diseases, such as cancer, multiple sclerosis, and Alzheimer’s disease [48]. Antioxidants possess the capability to neutralize ROSs through two primary mechanisms: direct reduction via electron transfer and radical quenching via hydrogen atom transfer. These processes result in the formation of more stable species, effectively mitigating oxidative stress and preventing the subsequent cascade of tissues, cells, and DNA damage [36].

### 3.5. Reducing Power

Reducing power, which characterizes the ability of an antioxidant to donate electrons for its own oxidation in redox reactions, is both an important manifestation of the antioxidant activity of an antioxidant and a rational explanation of its antioxidant capacity [49].

**Figure 4:**
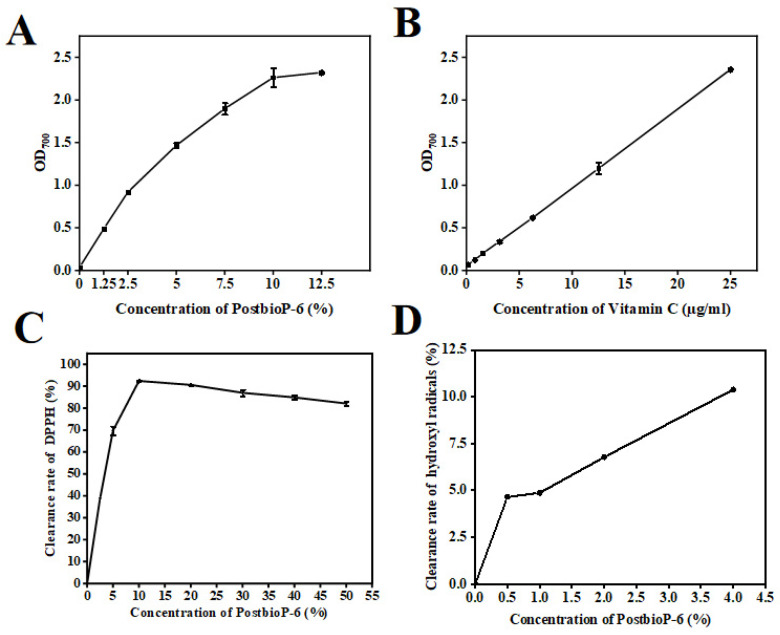
Antioxidant activity of PostbioP-6. (**A**) Reductive capacity of PostbioP-6. (**B**) Reductive capacity of VC. (**C**) DPPH free radical scavenging activity of PostbioP-6. (**D**) Hydroxyl radical scavenging activity of PostbioP-6. Data are the mean ± SD of three replicates.

In this reducing power detection assay, the reducing agent reduces the Fe^3+^/ferricyanide complex to the ferrous form, which can be monitored by measuring the formation of Prussian blue that can be measured at an absorbance of 700 nm [50]. Our results showed that PostbioP-6 exhibited a strong reducing power that progressively increased with a higher concentration (Figure 4A). The polynomial regression analysis demonstrated that the relationship between the concentration of PostbioP-6 and its reducing power fit the equation Y = d + B_1_ × X + B_2_ × X^2^ (d = 0.06176 ± 0.03559, B_1_ = 0.35484 ± 0.01502, B_2_ = −0.01387 ± 0.00118; R^2^ = 0.99). In particular, the reducing power of 12.5% PostbioP-6 is comparable to that of 25 μg/mL vitamin C. It was reported that the extracellular polysaccharide (EPS) from the endophytic bacterium *Paenibacillus polymyxa* EJS-3 exhibited notable reducing ability. Interestingly, the crude EPS had a higher reducing ability than the purified fractions. This is likely because some other antioxidant components, such as proteins, amino acids, peptides, and organic matter, might be present in the crude EPS [51]. Although the mechanism of PostbioP-6 is not fully understood, it is known that some natural antioxidants can act as electron donors, reacting with free radicals to convert them into more stable products and, thereby, terminating the free radical chain reaction [46].

### 3.6. DPPH Free Radical Scavenging Activity

DPPH, a stable free radical, is one of the most commonly used agents for the evaluation of the antioxidant capacity of a substance in vitro. In the assay, after the DPPH radicals are removed by the antioxidants, the reaction mixture changes color from purple to yellow, leading to a decrease in the absorbance at 517 nm [52]. Our results showed that the DPPH free radical scavenging capacity of PostbioP-6 was dose-dependent (Figure 4C). At a concentration of 10%, the scavenging capacity reached a maximum value of 92.4%, demonstrating the strong DPPH free radical scavenging potential of PostbioP-6. Furthermore, it was shown that the relationship between the concentration of PostbioP-6 and the DPPH free radical scavenging activity fit the generalized linear logistic model [Y = A_2_ + (A_1_ − A_2_)/(1 + (X/X_0_)^p^, A_1_ = 0.21924 ± 5.36345, A_2_ = 87.52929 ± 2.6683, X_0_ = 2.76406 ± 0.27352, p = 2.71632 ± 0.75465; R^2^ = 0.97].

A kind of postbiotic, named postbiotic RG14, prepared from a *Plant yeast* RG14 fermentation cell-free supernatant was proven to exhibit high antioxidant activity against DPPH free radicals and also to enhance glutathione peroxidase (GPX) activity in vivo [53]. Another type of postbiotic, derived from the co-fermentation of *Bacillus amyloliquefaciens* J and *Lactiplantibacillus plantarum* SN4, exhibited a DPPH scavenging capacity comparable to that of ascorbic acid [54]. Therefore, postbiotics may be a potent antioxidant with significant application potential in medicine, healthcare products, and functional foods.

### 3.7. Hydroxyl Radical Scavenging Activity

Hydroxyl radicals are the most reactive oxygen radicals in biological cells, causing oxidative damage to tissues and cells, leading to aging and chronic inflammation [55,56]. Similar to its DPPH free radical scavenging activity, PostbioP-6 exhibited dose-dependent hydroxyl radical scavenging capacity (Figure 4D). In addition, it was shown that the relationship between the concentration of PostbioP-6 and its hydroxyl radical scavenging activity fit the allometric model [Y = aX^b^, a = 5.33856 ± 0.47767, b = 0.45524 ± 0.08363; R^2^ = 0.94]. At a concentration of 4%, the hydroxyl radical scavenging capacity reached 10.38%, suggesting substantial scavenging potential. However, the maximum scavenging capacity was not achieved at this concentration, indicating the robust hydroxyl radical scavenging ability of PostbioP-6.

Jang et al. reported that intracellular extracts of *Lacticaseibacillus plantarum* exhibited high antioxidant activity in the concentration range of 10^8^ to 10^10^ CFU/mL, with certain dose-dependence and strain-specificity, and a strain of *L. plantarum,* named *L. plantarum* C88, had the strongest scavenging ability of hydroxyl radicals, with a scavenging rate of 44.31% at a cell concentration of 10^10^ CFU/mL. Additionally, Li et al. demonstrated that the EPS from *Lacticaseibacillus helveticus* MB2-1 exhibited potent scavenging activity against hydroxyl radicals. And at a concentration of 4 mg/mL, the crude EPS showed a hydroxyl radical scavenging rate of 80.24%, surpassing that of its purified fraction. This difference is likely due to the presence of other antioxidant components, such as proteins, peptides, and trace elements, in the crude EPS. One of the mechanisms of its hydroxyl radical scavenging capacity might be due to the active hydrogen-donating capacity of hydroxyl substituents in EPS [57].

### 3.8. Antimicrobial Activity

The Clinical and Laboratory Standards Institute (CLSI) guidelines were used to evaluate the drug sensitivity pattern of PostbioP-6 against different bacteria and fungi. A zone of inhibition greater than 20 mm indicates extreme sensitivity. Zones measuring 15–20 mm are considered highly sensitive, 10–14 mm moderately sensitive, and less than 10 mm hypoallergenic. A zone of 0 mm indicates resistance. The results show that PostbioP-6 exhibited a different degree of antimicrobial activity against 15 indicator bacteria and two fungi (Table 1): strong inhibitory activity against *S. aureus*, *Y. enteritis,* and *E. coli*; moderate inhibitory activity against *L. monocytogenes*, *S. typhimurium*, *P. fluorescens*, *P. putida*, *P. fragi,* and *E. sakazakii*; weak inhibitory activity against *P. lundensis* and *A. flavus*; and no inhibitory activity against *B. cereus*, *P. aeruginosa*, *P. citri,* and some common LAB and/or probiotic bacteria, such as *L. plantarum* P-8, *L. rhamnosus* Probio-M9, and *L. paracasei* Zhang.

Significantly, PostbioP-6 exerted good antibacterial activity against several food spoilage bacteria, such as *P. fluorescens, P. putida,* and *P. fragi*, indicating that PostbioP-6 has the potential to be applied in food preservation. In addition, PostbioP-6 exhibited no inhibitory effect on LAB or probiotics, making it a desirable food biopreservative that might not inhibit endogenous beneficial LAB and probiotics in the gut when applied to food.

Many studies have proved the antibacterial activities of postbiotics. For example, Kareem et al. reported that cell-free supernatants from *Lacticaseibacillus plantarum* strains RG11, RG14, RI11, UL4, TL1, and RS5 had significant inhibitory activity against a variety of pathogenic bacteria, including *L. monocytogenes* L-MS, *Salmonella* spp. S-1000, *E. coli* E-30, and vancomycin-resistant enterococci [58]. A kind of postbiotic from *L. casei* significantly inhibited the formation of virulence factors and biofilms, such as *Pseudomonas aeruginosa* pusillin and rhamnolipids, and effectively reduced the virulence attack and adhesion of harmful bacteria [59]. Some of the biosurfactants from probiotics had antimicrobial and anti-biofilm effects, disrupting biofilm integrity, leading to cytoplasmic loss, and preventing bacterial and fungal colonization [60]. Biosurfactants produced by *Lactococcus lactis* and *L. plantarum* were found to interfere with *S. aureus* biofilm formation and slightly inhibit *S. aureus* growth [61]. The antibacterial activity of postbiotics is primarily due to their rich content of various antibacterial components, such as bacteriocins, antibacterial peptides, exopolysaccharides, and short-chain fatty acids [62,63], and the variation in the antibacterial activity and antibacterial spectrum is attributed to the different composition of these components.

### 3.9. The Effects of PostbioP-6 on the Peroxide Value and Malondialdehyde Content in Cookies

**Figure 5:**
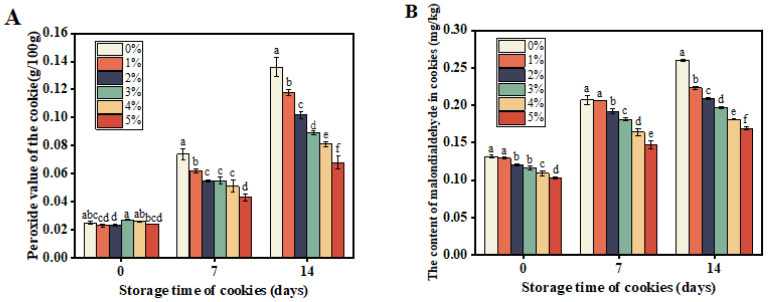
Changes in peroxide values and malondialdehyde content in cookies during storage. (**A**) Peroxide value in cookies with various concentrations of PostbioP-6 during storage. (**B**) Malondialdehyde content in cookies with various concentrations of PostbioP-6 during storage. Different letters (a–f) indicate statistically significant differences (*p* < 0.05) between groups and the data are the mean ± SD of three replicates.

The peroxide value serves as a common indicator to gauge the extent of lipid oxidation in food. The generation of malondialdehyde in food typically arises from chemical reactions involving fats, proteins, and carbohydrates, influenced by elevated temperatures, oxygen exposure, and microbial activity. As depicted in Figure 5A, the peroxide values increased in all the samples during storage. However, compared to the control (no PostbioP-6), the increase in the peroxide value in the cookies with PostbioP-6 added was significantly inhibited. Moreover, higher concentrations of PostbioP-6 resulted in a more pronounced decrease in the peroxide value. Similarly, the malondialdehyde content in the cookies spiked with various concentrations of PostbioP-6 exhibited a similar trend to the changes in the peroxide value (Figure 5B). These findings suggest that PostbioP-6 can attenuate food spoilage by inhibiting oxidation and malondialdehyde formation, underscoring its potential application in food preservation.

## 4. Conclusions

The present study prepared a postbiotic, named PostbioP-6, from *L. paracasei* Postbiotic-P6 and investigated its multifaceted activities, including anti-inflammatory, antihemolytic, antioxidant, and antimicrobial activities. Its multifunctional capabilities suggest its potential application in various health domains, such as protection against multiple diseases, reduction of oxidative stress in tissues, and inhibition of foodborne pathogens and spoilage bacteria. This study will help to promote in-depth research in and the application of postbiotics. In the future, further research is needed to elucidate postbiotics’ precise mechanisms of action, identify the active components responsible for these functions, and validate the health benefits in animal models or clinical trials.

## Figures and Tables

**Table 1:** Inhibitory spectrum of PostbioP-6.

Strain Name	Form	Antibacterial Activity
*Staphylococcus aureus*	G+	+++
*Listeria monocytogenes*	G+	++
*Bacillus cereus*	G+	−
*L. paracasei*	G+	−
*L. rhamnosus*	G+	−
*L. plantarum*	G+	−
*Yersinia enterocolitica enteritis*	G−	+++
*Salmonella typhimurium*	G−	++
*Escherichia coli*	G−	+++
*Pseudomonas fluorescens*	G−	++
*Pseudomonas putida*	G−	++
*Pseudomonas aeruginosa*	G−	−
*Pseudomonas fragi*	G−	++
*Pseudomonas lundensis*	G−	+
*Enterobacter sakazakii*	G−	++
*Aspergillus flavus*	fungi	+
*Penicillium citri*	fungi	−

Remarks: “+++”, “++”, and “+” represent highly sensitive, moderately sensitive, and hypoallergenic, respectively, “−” means no inhibition.

## Data Availability

The original contributions presented in the study are included in the article, further inquiries can be directed to the corresponding author.
